# The Best Closure Technique Without Mesh in Elective Midline Laparotomy Closure

**DOI:** 10.3389/jaws.2022.10962

**Published:** 2022-12-07

**Authors:** René H. Fortelny

**Affiliations:** ^1^ Department of General, Viszeral and Oncologic Surgery, Wilhelminenspital, Vienna, Austria; ^2^ Faculty of Medicine, Sigmund Freud Private University Vienna, Vienna, Austria

**Keywords:** elective midline closure, small bites, large bites, incisional hernia prevention, stitch technique

## Abstract

**Introduction:** The risk of developing an incisional hernia after primary elective median laparotomy is reported in the literature as being between 5 and 20 percent. The basic of an optimal outcome after midline incision is the appropriate closure technique with or without a prophylactic mesh. The objective of this paper is to critically examine the various closure techniques and, in particular, to present a detailed comparison of the long stitch and short stitch techniques.

**Method:** Based on the available literature, the characteristics of the different closure techniques are described in detail, advantages and disadvantages are compared, and the current status of a practicable recommendation is discussed. Special attention is paid to the criteria of the short stitch technique, such as the suture to incision length ratio, number of stitches and distances, as well as suture material.

**Results:** For elective midline closures, the use of a continuous closure using a slowly absorbable suture material in the small-bites technique with suture to wound ratio of at least 5:1 result in significantly lower risk of complications such as bursting abdomen and less incisional hernia rates compared to the large-bites technique.

**Conclusion:** Based on the present evidence in midline closure after elective laparotomy the small bites technique can be recommended to significantly reduce the rate of incisional hernia.

## Introduction

Despite the advancement of minimally invasive techniques in visceral surgery, conventional midline laparotomy remains the standard approach for major surgery as well as emergency procedures. Over the past decades, there has been debate about the best possible closure technique and the suture material to be preferred. After the review by Diener et al. (1) in 2010, it was evident that the continuous suture technique with long-term absorbable suture is to be preferred in elective midline closure. Analogous to Diener’s review the published Cochran review of 2017 (2) summarized that monofilament sutures can be considered for abdominal closure to reduce the risk of incisional hernia and absorbable sutures can be considered to reduce the risk of chronic drainage from the wound. However, due to the lack of evidence, these reviews did not include a discussion or recommendation regarding the stitching technique with small or large bite. In 2017, the MATCH review by Henriksen et al. (3) followed, which included the randomised controlled trials by Millbourn et al. (4) and the STITCH trial (5) in a subgroup analysis. The cumulative incisional hernia rate for the small bite technique has been significantly lower at 9.45% compared to 19.30% for the large bite technique (*p* = 0.005, OR 0.41; 95% CI 0.19, 0.86). The conclusion from this review to be drawn is that using a slowly absorbable suture material and a continuous suture technique with small tissue stitches lead to a significant reduction in the incisional hernia rate compared to a technique with large stitches. The recently published update of the EHS guidelines for closure of the abdominal (6) based on two RCT studies (4,5) include only one strong recommendation regarding the suture technique to use a continuous suture technique in elective midline closure. All other topics, like small or large bite technique, suture material, were graded with a weak recommendation due to the lack of high evidence based on GRADE recommendation (7). Now, however the recently published data of the ESTOIH study (8,9) are available and might change the evidence in some degree. In the short-term results (8) a significantly lower risk for burst abdomen was found in the cox proportional hazard model [HR 0.1783 (0.0379–0.6617), *p* = 0.0115] after short bite technique. The incisional hernia rate after 1 year (9) revealed 4.24% after small bite and 8.23% after large bite technique (*p* = 0.14%). Although the difference was not significant, the results were significantly better compared with the Millbourne and STITCH study. Even if the prevention of potential sources of complications is to be seen as multifactorial, at least the surgical closure technique as a standardized procedure remains an essential factor for an uncomplicated wound healing of the abdominal wall.

However, even given that the short stitch technique seems to be evident, the technique is still slightly different in the three studies mentioned above. In addition to the suture technique, the suture material used in combination with the needle size, shape and thickness is another important factor. Standardization is therefore an essential issue to achieve comparability of studies in the future (10). Moreover, the greatest risk factor for an uncomplicated course of midline closure, among many other factors, still seems to be the surgeon himself (11).

### Opening of the Midline

To achieve the best possible conditions for abdominal wall closure, an exact midline opening is essential. This implies that the crossing fiber bundles of the linea alba should be targeted as centrally as possible, i.e., at the crossing point, and thus the integrity of the linea alba should be preserved. Only in this way the anchoring of the suture in the aponeurotic tissue is ensured during suture closure. The safest landmark for the start of the incision is the umbilical ridge, which after detachment reveals a natural opening that represents the exact midline of the linea alba. Therefore, the incision should always be performed at this point. Another criterion is the detachment of subcutaneous fatty tissue in front of the linea alba or anterior sheath of the rectus muscle before opening over a distance of 1 cm on both sides, as well as cranially and caudally. Only then the crossing fibers of the linea alba are invisible and an exact midline incision can be safely performed without splitting the anterior and posterior sheath. This special technique was also a crucial part of the ESTOIH study protocol (12).

### Experimental Background of Closure Techniques

The small bite technique was first investigated experimentally by Israelsson and his scientific group in 2001. In this experimental study published by Cengiz (13) the advantages of the small bite technique in terms of bursting strength compared to the long bite technique could be demonstrated significantly. The burst strength after small bites technique was 3-fold higher than after the large bites. Harlaar also impressively highlighted the advantages of the short-stitch technique in his experimental study (14). The so-called slacking effect was demonstrated with the large bite technique in the all-in-one stitch version. This could be avoided by including only the fascia without muscle tissue. As early as 2000, Höer and his team demonstrated the importance of tensile loading of the suture closure regarding blood supply, deposition of mature collagen and scar healing (15,16,17,18). The slacking effect has also been described here, which can be avoided by reducing the tensile load on the suture line. These experimental studies have also pointed out the advantage of the continuous suture over the interrupted suture technique.

### Suture Tension

The problem of surgeon control of suture tension remains difficult. The direct correlation of suture tension, blood flow and wound healing has been experimentally demonstrated by Höer et al. (19). Based on the studies on the tensile strength of the intact linea alba by Hollinsky et al. (20), a maximum horizontal traction of 10 N corresponding to 1 kg tensile load is possible. This value decreases by 30% in the case of a scar after laparotomy. Thus, it seems clear that the tensile load applied to the suture line should not exceed 1 kg. This limit is confirmed in the clinical works of Klein et al. (21) and Dragu et al. (22) in relation to the choice of procedure for incisional hernias. Therefore, verification of the applied suture tension seems to be an absolute problem. A study by Höer et al. (23) and Schachtrupp et al. (24) measured suture tension under simulation of fascial closure. The results of the surgeons involved were sobering regarding the specifications and reproducibility. The conclusion of this study was that it is hardly possible to meet the target values without measuring the suture tension (tensiometer).

The experimental study of Klink et al. (25) in a rodent model obtained that non-elastic monofilament sutures rapidly loose tension independently of the sutured tissue. Based on these results high tension seems to be associated by the force to the sutures by the surgeon. This hypothesized approach of reduced tissue compression resulting in less local tissue damage, thus achieving improved wound healing, is a fundamental part of preventing complications by the surgeon himself. A direct indication of low suture tension is the recommendation of an adaptive traction at the suture line, as recommended by Israelsson and implemented in the protocol of ESTOIH study (12).

**FIGURE 1:**
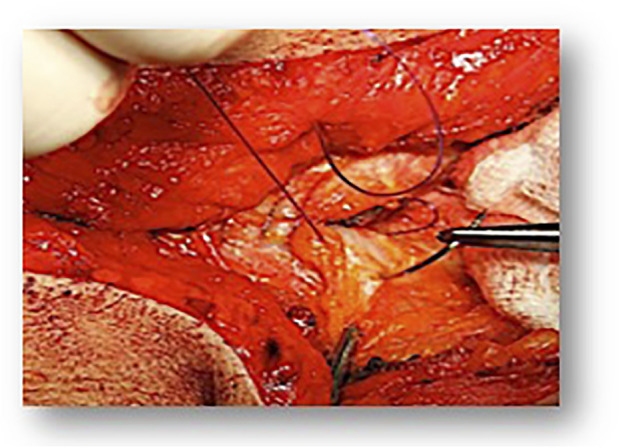
Small bites technique.

If the suture tension of the fascial suture plays a decisive role for an undisturbed healing process, the surgeon should be able to determine the tension of the fascial suture with the aid of, e.g., tensiometry or, in a simplified way, by the indicator of the visible suture bridges after completion of the closure (Figure 1). Another aspect is a continuous suture technique over the entire incision without interrupting suture ties. This ensures undisturbed suture tension on the entire suture closure according to abdominal compliance. Therefore, sufficient suture length must also be considered in order to be able to perform the entire closure suture with one suture material. These requirements were met in the ESTOIH study with the use of a 150 cm long suture material. The frequently used technique with two sutures simultaneously from the cranial and caudal sides with knotting in the middle of the joining sutures must therefore be considered critically.

### Needle Size, Needle Diameter and Suture Size

**FIGURE 2:**
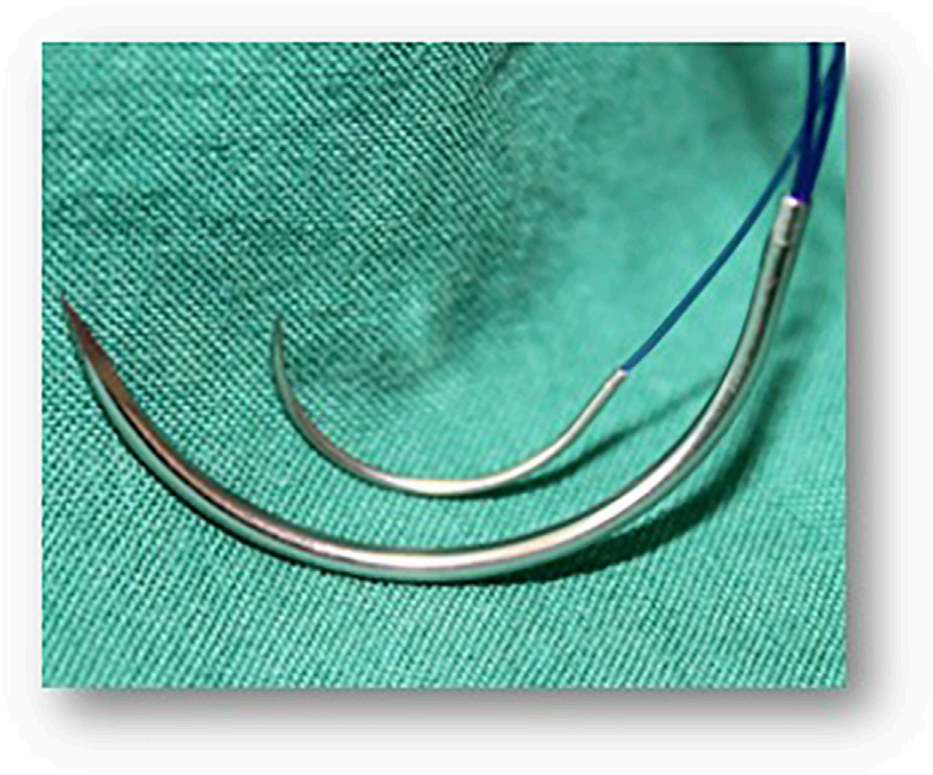
Suture/Needle: Large bites technique: Suture MonoMax® USP 1, 150 cm loop, Needle HR 48 Small bites technique: Suture MonoMax® USP 2-0, 150 cm, Needle HR 26.

The size and especially the diameter of the needle used is directly related to the stitch defect set around the fascia. For this reason, the use of a loop suture with a resulting large calibre needle (e.g., HR 48) is always associated with a large defect in the tissue (Figure 2). Since the publications of Israelsson, the use of small size and diameter needles (e.g., HR26) has become common in the short stitch technique. The size of the suture material used is usually 0 or 1 for loop sutures but should be preferably 2/0 for the short stitch technique. The tensile strength of a suture is still very often associated by surgeons with the thickness of the suture and the technique of long stitches or even interrupted sutures. Although the rate of burst abdomen did not differ significantly between the short and long suture techniques in the Millbourn and STITCH studies, the hazard ratio in the ESTOIH study showed a 7-fold reduction in the risk of developing a burst abdomen when the short suture technique was used.

### Suture to Wound Length Ratio

First, one must consider that the SL-WL ratio, cannot be clearly defined, since any ratio can be achieved by varying either the tissue bites or the distances or the intervals between the stitches, or both variables. These variations will result in an endless number of ways to achieve this ratio. Therefore, only the precise definition of the number of stitches, the stitch distances, the suture material consumption and the resulting specification of the SL-WL ratio together can enable a verifiable standardization of a short stitch technique (Table 1).

**TABLE 1:** Small bites technique in detail.

“Small bites”-technique:
▪ Suture material: monofilament, elastic, slowly absorbable
▪ Size: 2/0
▪ Continuous suture technique
▪ Only fascia including
▪ Suture/wound-length ratio ≥ 5:1
▪ First stitch distance to incision > 1 cm
▪ Stitch to incision 5–8 mm
▪ Stitch to stitch: 4–5 mm
▪ Stitch length ≤ 2,5 cm
▪ Adaptive suture tension (≤1 kp)
▪ Cave > “button holes"
▪ Visible suture bares

Höer et al. (15) demonstrated in experimental studies that the SL-WL ratio, suture tension and suture technique have been shown to have a significant influence on the mechanical strength of the incision. Small bites closures with a SL-WL ratios of 4:1 and 8:1 led to the highest tensile strength after 14 days (mean 20.99 ± 3.24 N/cm and 19.62 ± 1.47 N/cm, respectively). The importance of low tension on the suture line resulted in significantly weaker scars, regardless of the suturing technique used. In agreement with clinical data, it could be experimentally demonstrated that running closure of midline laparotomies with a SL-WL ratio above 4:1 while avoiding high suture tension had a significant positive effect on the mechanical strength of the incision.

In an experimental study in pigs, Kushner et al. (26) were able to demonstrate the benefits of blood perfusion in small bite closure already demonstrated by Höer et al. (15). In this study, in addition to small and large bites with PDS suture, a barbed suture (Stratafix™) using the same techniques and additionally an interrupted figure of 8 with PDS were examined by laser-induced fluorescence angiography regarding tissue perfusion immediately after closure and 1 week later. The results revealed a significant increase in tissue perfusion after small bite closure with PDS suture. In contrast, neither the interrupted figure of 8 nor the barbed suture significantly increased tissue perfusion at 1 week. Consequently, it seems that there is no advantage for midline closure with either the figure of 8 interrupted or the barbed suture technique. In a prospective study by Israelsson et al. (27) the suture length to wound length ratio <4 was identified as independent risk factor for the development of incisional hernia in comparison to ≥4 (23.7% versus 9%; *p* = 0.001).

### Impact of Suture Material

After the stitch and suture technique, the suture material is certainly the decisive factor with regard to the stability of the fascial closure. Since the systematic review by Diener et al. (1) and the recently published updates of the guidelines for abdominal wall closure (5), the continuous closure technique by small bites technique with the use of a slowly absorbable suture material is recommended. Consequently, the use of a monofilament suture material is also to be preferred. When assessing the quality criteria, the bursting strength of the suture material is erroneously used as an essential quality criterion. In view of the compliance of the abdominal wall and the associated stresses on the midline closure, it seems reasonable to use an equally elastic suture material for the continuous closure. In comparing the properties of the various suture materials that are preferably used for abdominal wall closure, Albertsmeier et al. (28) compared poly-4-hydroxybutyrate (MonoMax®) with polydiaxanone (PDS®, MonoPlus®) in their publication. The comparison regarding elongation (elasticity) detects a clear advantage of poly-4-hydroxybutyrate with 90% to a maximum of 50% for polydiaxanone. The basic strength retention of 50% up to 100 days is also significantly longer compared to 42 and 35 days for polydiaxanone. An additional criterion to be considered is the mass absorption time, which at 390 days is almost twice as long for poly-4-hydroxybutyrate as for polydiaxanone and thus supports wound healing over a long period. The increased elasticity can be expected to reduce suture tension, especially during a sudden increase in intra-abdominal pressure such as during coughing, weeping, or jumping. This mechanism potentially reduces reiterative injury to the rectus fascia, ultimately leading to burst abdomen and, in the long-term, to incisional hernia. In another study, France et al. (29) was able to demonstrate that a viscoelastically active suture can accelerate wound healing due to a significant increase in the motility of human fibroblasts and thus lead to improved scar formation.

### Clinical Evidence

To date, three randomized controlled trials have been published on the short versus long stitch technique in midline laparotomy (4,5,9). Although the short stitch technique seems not to differ significantly in the protocols in these studies, the 1-year outcome between the Millbourn study, the STITCH-trial and the ESTOIH study is markedly different with 5.6% versus 13% versus 4.23% regarding the incisional hernia rate (Table 2). Obviously, there must be a specific cause behind, which is extremely complex to analyse retrospectively.

**TABLE 2:** Incisional hernia rate in comparison Millburn-, STITCH- and ESTOIH study.

Technique	Incisional hernia		
	MILLBOURN	STITCH	ESTOIH
Long stitch	18%	21%	8.23%
Short stitch	5.6% sign.	13% sign.	4.24% n.sign.

**FIGURE 3:**
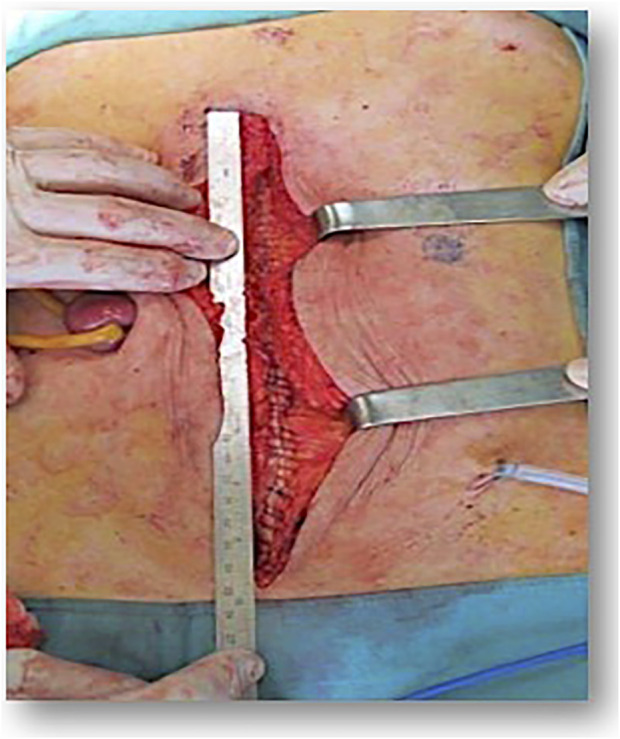
Small bites closure with ruler.

**FIGURE 4:**
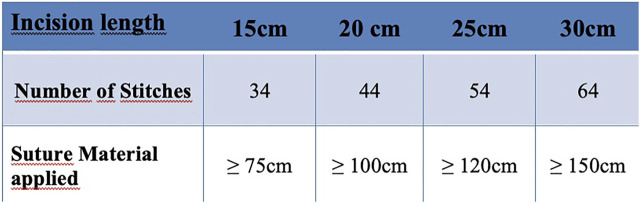
Small bites: Suture to Wound Length - Ratio (≥5:1) Example: incisional length, number of stitches, applied suture material.

An important parameter for a specific analysis could be the ratio of suture to wound length in the short stitch technique group. Even though this ratio is not an absolute value for the exact performance of a short stitch technique, since the ratio of used suture material to incision length ultimately only provides an indirect measure depending on the number of stitches, stitch distance and circumference, it is still the most important parameter for the closure technique that needs to be recorded (Figures 3, 4). When comparing this ratio across the three studies, the highest value for short stitch technique was 5.7 in the Millbourn study, followed by 5.3 in the ESTOIH study and 5.0 in the STITCH study. These differences may seem small at first sight but could be related to the significantly different incisional hernia rates. As Israelsson has clearly demonstrated the importance of this ratio in several studies (27,30), the lowest limit for this ratio seems to be above 4:1 for the short stitch technique.

A further essential factor could be the properties of the suture material, as previously mentioned. In the Millbourn study and the STITCH study, an identical suture material made of polydiaxanone (PDS^©^) was applied. In the STITCH study, a polydixanone with triclosan coating (PDS plus^©^) was apparently used to reduce infection complications. The different infection rates of the two studies, especially the high infection rates of more than 20% in both groups in the STITCH trial, are difficult to interpret, but the relationship between infection and risk of incisional hernia must be considered (Supplementary Material).

During the ESTOIH study, the poly-4-hydroxybutyrate (Monomax^©^) suture material was used, which differs significantly from the polydiaxanone material. The high elasticity of this suture material in combination with an ultra-long resorption time are criteria that appear to be beneficial in midline closure. A synergistic component of suture technique in small bite and the elasticity of the suture material can provide healing of the midline closure supported over a longer period of time, resulting in a stable scar. According to the data of the ESTOIH study, the choice of suture material seems to have a potential effect by using a highly elastic and ultra-long-lasting resorbable material. However, the adequately applied suture technique is a basic prerequisite for a complication-free outcome.

## Standardisation, Training and Implementation

One of the most important steps in implementing a new surgical method is standardization. As in many examples of visceral surgery, e.g., Shouldice plasty, the modification of a surgical method not only leads to different results, but also prevents a scientific comparison between them. Therefore, the exact definition of the individual surgical steps is of enormous importance. In the case of the short stitch technique for elective midline closure, standardization begins with the performance of the median laparotomy, as described in this review. The best possible closure can only be achieved and guaranteed after the best possible midline incision has been made. Therefore, a protocol for elective midline closure should include the incision technique. During the ESTOIH study, this important part was described in detail and communicated and trained with all study canters before the start of the study.

Conway et al. (31) demonstrated that neither trainee nor surgeons are able to estimate the distances recommended in small bite technique with accuracy. Therefore, the need of surgical training to achieve such skills is fundamental.

An experimental study by Lesch et al. (10) impressively demonstrated the advantages of standardizing defect closures in short- and long-term techniques. Various parameters regarding the durability of a repair were used. The strongest significant improvement was demonstrated by standardizing the suture technique.

In a study by Pereira Rodrigez (32), after hands-on training on a suture simulator model with the participation of 74 surgeons, a survey was conducted after 1 year to evaluate the implementation of the short-stitch technique in elective midline laparotomy. Of 114 median laparotomies, 30.7% were performed using the short-stitch technique, which had a lower incisional hernia rate of 3.6% versus 12.1% compared to the long-stitch technique. Nevertheless, despite hands on training, the implementation seems to be poor without further incentives. Another study by Thorup et al. (33) reported that following the introduction of a standardized small bites technique in acute midline laparotomies, the incidence of burst abdomen decreased from 5.6% to 2.2% and the incidence of incisional hernia declined from 27% to 15% after 2 years of follow-up compared to a historical cohort using different closure methods. Both studies clearly highlight the importance of standardization and consistent implementation to reduce incisional hernias.

## Discussion

Median laparotomy remains the standard approach in open visceral surgery and is associated with high rates of incisional hernia. In summary of the existing literature, the short stitch technique is significantly superior to the long stitch technique and should therefore be implemented in a standardized technique. This requires training and feedback to avoid technical errors and slow learning curves with complications such as suture rupture and burst abdomen. The time required for closure using the short-stitch technique must not and should not be an argument against this procedure. As can be seen from the studies, this is an investment of 5–6 min compared to the long stitch technique, which has no relation to the follow-up costs in case of complications or even the repair of a incisional hernia. The cost-benefit analyses as described by several authors (34,35,36) clearly show the advantage of the short stitch technique. Irrespective of the costs related to the treatment of incisional hernias, the personal fate of the patient must be considered and taken as an important factor. The established risk factors for the development of incisional hernias, such as the presence of a collagen metabolic disorder, BMI >27, AAA, and other comorbidities, should be considered in any laparotomy and will influence the closure procedure. The use of prophylactic mesh procedures is increasingly discussed and recommended for these risk factors (6). The evidence on these procedures is based primarily on the significant results of numerous studies that have followed closure exclusively with long stitch procedures (37,38,39). Therefore, in the future, as already implemented in ongoing studies, a short stitch procedure should always be used as the basis for this mesh-augmented closure. Thus, every midline laparotomy, regardless of risk factors, should be closed using a short stitch technique as a matter of principle in order to sustainably reduce the scar hernia rate in the future.

The universal introduction of the short stitch technique, as with many new surgical procedures, cannot be communicated solely by publications, but only by offering workshops and training courses as a standardized procedure (31,32,33). Even in the setting of emergent laparotomies, short stitch techniques have an immediate and impactful effect on reducing complications (33).

Thus, the biggest challenge remains to disseminate the short stitch technique in a standardized technique and to implement it not only in open visceral surgery, but also in gynecological, urological and vascular surgery.

## Conclusion

In the summary of the existing literature, the short stitch procedure should be considered the standard procedure for closure after elective midline laparotomy to reduce the incisional hernia rate. Only appropriate standardization and teaching of this technique by means of training can ensure widespread implementation of this method in the midterm.

## Supplementary Material

Supplementary Image S1 SSI rate in comparison Millburn-, STITCH- and ESTOIH study.
