# Supplementary figures and images for: The Best Closure Technique Without Mesh in Elective Midline Laparotomy Closure
Source: J Abdom Wall Surg. 2022 Dec 7;1:10962. doi: 10.3389/jaws.2022.10962 (PMC10831662; doi:10.3389/jaws.2022.10962)

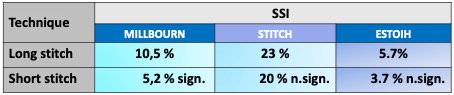

Supplement: Supplementary file 1 [file Image1.jpeg]
